# Association between Left Atrial Appendage Morphology and Clot Histology in Patients with Embolic Ischemic Stroke: An Exploratory Study

**DOI:** 10.3390/jcm13061734

**Published:** 2024-03-17

**Authors:** Givi Lengvenis, Julius Drachneris, Edvardas Žurauskas, Aleksandra Ekkert, Andrius Berūkštis, Marius Kurminas, Rokas Girčius, Kipras Mikelis, Andrej Afanasjev, Kristina Ryliškienė, Arvydas Laurinavičius, Algirdas Edvardas Tamošiūnas

**Affiliations:** 1 Department of Radiology, Nuclear Medicine and Medical Physics, Institute of Biomedical Sciences, Faculty of Medicine, Vilnius University, 03101 Vilnius, Lithuania; marius.kurminas@santa.lt (M.K.); rokas.gircius@santa.lt (R.G.); kipras.mikelis@santa.lt (K.M.); andrej.afanasjev@santa.lt (A.A.); algirdas.tamosiunas@santa.lt (A.E.T.); 2 Department of Pathology and Forensic Medicine, Institute of Biomedical Sciences, Faculty of Medicine, Vilnius University, 03101 Vilnius, Lithuania; julius.drachneris@vpc.lt (J.D.); edvardas.zurauskas@vpc.lt (E.Ž.); arvydas.laurinavicius@vpc.lt (A.L.); 3 Clinic of Neurology and Neurosurgery, Institute of Clinical Medicine, Faculty of Medicine, Vilnius University, 03101 Vilnius, Lithuaniakristina.ryliskiene@santa.lt (K.R.); 4 Clinic of Cardiac and Vascular Diseases, Institute of Clinical Medicine, Faculty of Medicine, Vilnius University, 03101 Vilnius, Lithuania; andrius.berukstis@santa.lt

**Keywords:** stroke, LAA, embolic, histopathology, mechanical thrombectomy

## Abstract

Background: Acute embolic ischemic stroke poses a significant healthcare challenge. Histological clot features’ variability among patients with acute ischemic stroke treated by mechanical thrombectomy has potential implications for determining treatment and etiology. This study investigated the clot histological feature differences among patients who experienced cardioembolic stroke and embolic stroke of undetermined source with different left atrial appendage (LAA) morphologies. Methods: We conducted a prospective observational study involving 79 patients with acute embolic ischemic stroke undergoing mechanical thrombectomy. Computed tomography angiography images were used to classify LAA morphologies. An artificial intelligence algorithm assessed the clot fibrin and red blood cell contents. Results: Patients with chicken-wing LAA morphology exhibited lower mean clot fibrin proportions than did those with non-chicken-wing morphology (*p* < 0.001). Linear regression analysis showed that chicken-wing LAA was significantly associated with a lower clot fibrin proportion (estimate, −0.177; 95% CI [−0.259, −0.096]; *p* < 0.001). The successful recanalization rate and first-pass effect between the groups did not differ significantly. Conclusions: The chicken-wing LAA morphological type is associated with lower clot fibrin contents, suggesting potentially different embolism mechanisms or diverse embolic sources, compared with the non-chicken-wing LAA types. Further studies are required to investigate this association.

## 1. Introduction

Embolisms are the dominant cause of acute ischemic stroke, and cardioembolic strokes as well as embolic strokes of undetermined source (ESUSs) are associated with the largest group of patients admitted for mechanical thrombectomy [1,2]. With widespread endovascular treatment use and histological investigations of the extracted clot, the substantial variability in histological clot features among patients with ischemic stroke has become apparent. These differences may have important implications in stroke treatment, etiology determination and secondary prevention [3].

Some studies have found diverse fibrin and red blood cell (RBC) proportions in clots between large-artery atherosclerosis and cardioembolic stroke. However, no significant difference has been observed between cardioembolic strokes and strokes of undetermined source [4,5]. Furthermore, existing data indicate that various morphologies of the left atrial appendage (LAA) exhibit diverse flow patterns and varying levels of stroke risk, with the chicken-wing type showing the lowest risk [6,7]. We hypothesized that the chicken-wing LAA is associated with a different clot histology compared to other LAA types. To test this hypothesis, we investigated clot histological features in different LAA morphologic types in patients with cardioembolic stroke and ESUS.

## 2. Materials and Methods

### 2.1. Patients

This prospective observational study was approved by the Vilnius Regional Bioethics Committee (No. 2022/2-1412-883, 15 February 2022). This study was conducted in accordance with the ethical principles of the Declaration of Helsinki. All participants signed a written informed consent form. All participant data were pseudonymized before being processed by the investigators.

**Figure 1:**
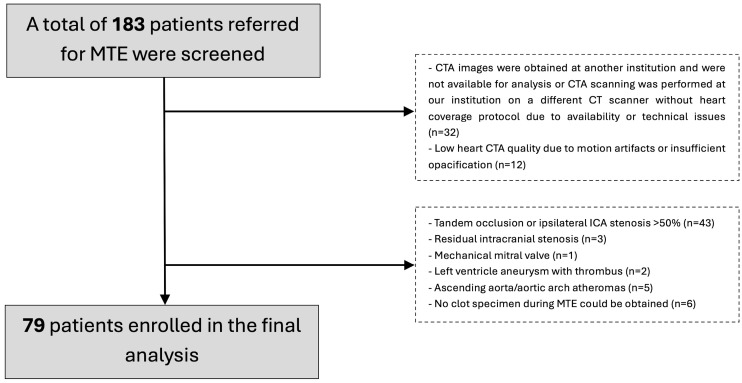
Flowchart representing patient selection. MTE, mechanical thrombectomy; CTA, computed tomography angiography; CT, computed tomography; ICA, internal carotid artery.

We enrolled 79 patients with acute ischemic stroke between March 2022 and June 2023 (a flowchart of patient selection is presented in Figure 1). The inclusion criteria were (1) age > 18 years, (2) receipt of mechanical thrombectomy treatment, (3) clot suitable for histological analysis and (4) availability of preprocedural computed tomography angiogram (CTA), covering the heart. The exclusion criteria were (1) tandem occlusion or extracranial artery stenosis > 50%; (2) any high-risk cardioembolic source besides non-valvular atrial fibrillation (AF) and LAA thrombus; (3) aortic atheromas in the ascending aorta, aortic arch or proximal descending aorta; (4) residual stenosis of the intracranial artery after thrombectomy at the initial occlusion; and (5) CTA motion artifacts or insufficient left cardiac chamber opacification impeding left atrial assessment. Patients with extracranial stenoses > 50% and intracranial stenoses, as well as aortic atheromas and other high-risk embolic sources, were excluded to minimize bias resulting from non-LAA clots.

An ESUS diagnosis was made according to our institution’s algorithm, based on Hart et al. [8]. The following criteria must be fulfilled to establish ESUS etiology: (1) non-lacunar stroke on computed tomography or magnetic resonance imaging; (2) absence of ipsilateral extracranial or intracranial artery stenosis >50% or signs of other arterial disease (dissection, arteritis, etc.) on CTA, magnetic resonance angiography, ultrasound examination, or digital subtraction angiography; (3) absence of AF on at least 24 h Holter monitoring; and (4) absence of cardioembolic sources on transthoracic echocardiogram. In case the described protocol failed to demonstrate a likely etiologic factor, we performed a contrast-enhanced transcranial Doppler ultrasound to investigate the presence of a persistent foramen ovale in patients under 60 years old. Additionally, for patients under 50 years old, we conducted a thrombophilia panel.

### 2.2. Imaging Protocol

All CTA images were acquired using a 128-slice computed tomography (CT) machine (Discovery CT750 HD; GE Healthcare, Milwaukee, WI, USA) with the following parameters: 120 kVp tube voltage, 350 mA tube current and 1.25 mm slice thickness. CTA images were reconstructed with a 0.625 mm slice thickness. The contrast agent used for CTA was Iohexol 350 mg I/mL (Omnipaque; GE Healthcare, Oslo, NO, USA) with a standard 100 mL dose (corrected if the patient size was exceedingly small or large) injected intravenously at 4 mL/s. A technologist manually activated scanning when the enhancement at the monitoring site reached 100 HU. The monitored region was in the left ventricular cavity.

As per the standard protocol at our institution (adapted from Popkirov et al. [9]), the lower limit of the CTA scan in the arterial phase was set at the diaphragm to include the heart and was scanned to the vertex. The venous phase was scanned from the base of the skull to the vertex.

### 2.3. CTA Analysis

LAA morphology assessment was performed by two experienced radiologists. The investigators were blinded to the clinical data and the histological results of the patients. The qualitative LAA morphology assessment was performed using the Advantage Workstation Server (version 3.2; GE Healthcare, Chicago, IL, USA). LAAs were divided into four types, as described by Wang et al. [10] and modified by Kimura et al. [11]: chicken-wing, windsock, cactus and cauliflower. The chicken-wing morphology had one dominant lobe with a minimum length of 40 mm and an angle less than 100° in the proximal part. The windsock morphology had a dominant lobe with a minimum length of 40 mm and an angle exceeding 100°. The cactus morphology had a dominant lobe of <40 mm with secondary lobes. The cauliflower morphology had a dominant lobe < 40 mm with no forked lobes. We opted for the classification proposed by Wang et al., as these morphologic groups are widely employed in studies examining the left atrial appendage (LAA). Additionally, the modification introduced by Kimura offers quantitative criteria, thereby reducing classification biases.

**Figure 2:**
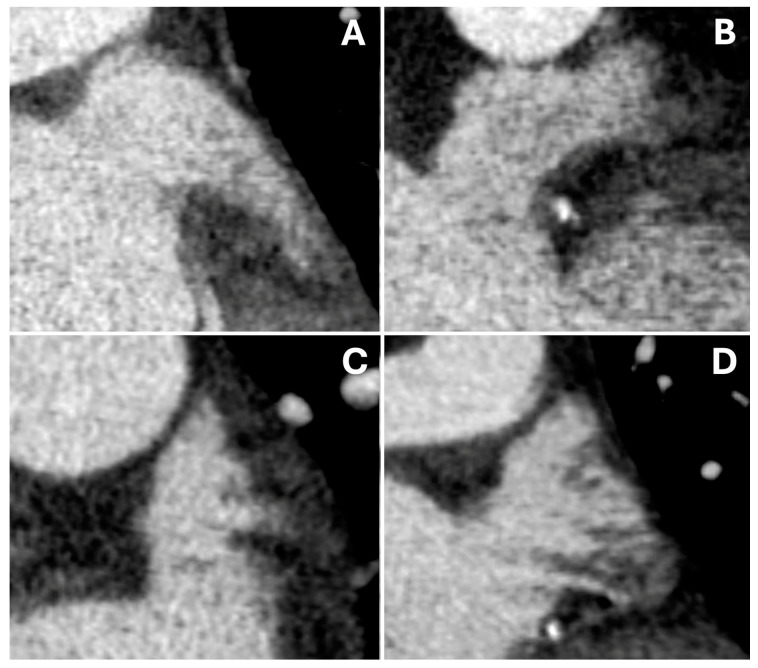
Representative examples of each left atrial appendage type on computed tomography angiography: (**A**) chicken-wing, (**B**) windsock, (**C**) cactus, (**D**) cauliflower.

Additionally, the left atrium and LAA were inspected for intraluminal thrombi, and the LAA was evaluated for circulatory stasis. Excellent inter-rater agreement for the LAA morphology classification was observed (kappa = 0.9). Any discrepancies between the two readers were discussed, and a consensus was reached. Figure 2 presents examples of each LAA type.

The left atrial and LAA volume measurements were performed using an open-source 3D Slicer platform (version 5.3.0; www.slicer.org) [12]. The left atrium and LAA cavities were manually segmented, and separate volumes of both structures were derived. The segment outline coincided with the LAA wall outline in LAA circulatory stasis cases.

Intracranial clot length was measured on CTA images using both arterial and venous phases for the best visualization of proximal and distal ends of the occlusion. The measurements were performed on multiplanar reconstructions.

### 2.4. Treatment

Patients suitable for mechanical thrombectomy after CT examination were referred to the Angiosuite. Mechanical thrombectomy was performed using a balloon guide catheter and stent retriever, or an aspiration catheter. The technique was chosen at the discretion of the operator. Final recanalization was reported using the Modified Thrombolysis in Cerebral Infarction (mTICI) score [13]. Successful recanalization was defined as a 2C/3 mTICI score. The first-pass effect was defined as a 2C/3 mTICI recanalization using the selected thrombectomy device after the first attempt.

The procedure was performed under local anesthesia and conscious sedation. General anesthesia was administered in cases where a patient had a Glasgow Coma Scale score ≤ 8 or experienced respiratory distress.

Bridging thrombolysis was administered to patients arriving within a 4.5 h window when the patient could not be promptly transported to the Angiosuite because of concurrent neuroradiology procedures or admission after working hours. A standard alteplase (0.9 mg/kg) dose was administered intravenously, with 10% as a bolus, and the remainder was continued as an infusion. After arrival at the Angiosuite, the remaining alteplase infusion was halted.

### 2.5. Histological Evaluation

All clot fragments of at least 1 × 1 mm in size were referred for histological analysis. After extraction, fresh clots were fixed in 4% phosphate-buffered formaldehyde and transported for pathological examination. After gross examination, the tissues were embedded in paraffin, sectioned at 3 micrometer thickness, and stained with hematoxylin and eosin (H&E). The pathologist performing histological analysis was blinded to the clinical and procedural data.

**Figure 3:**
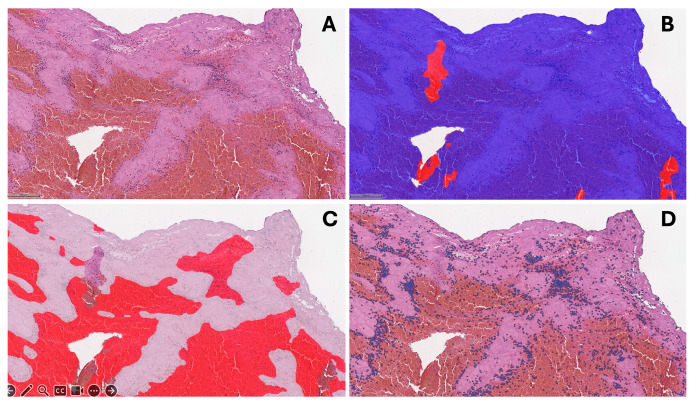
Clot histologic analysis process: (**A**) hematoxylin and eosin staining of representative thrombus tissue sample; (**B**) quality control slide V2 (HALO AI) tissue annotations—red represents poor quality (out of focus, overlapping section), blue—good quality; (**C**) Densenet v2 (HALO AI) tissue classifier annotation on good quality tissue—white represents fibrin-rich areas, red—erythrocyte-rich areas; (**D**) cell segmentation annotations (HALO AI).

We digitized H&E slides using an Aperio^®^ AT2 DX scanner (Leica Aperio Technologies, Vista, CA, USA) at 20× magnification. We used HALO^®^ AI (version 3.5; Indica Labs, Albuquerque, NM, USA) software for image analysis. We removed areas with artifacts using the QC slide V2 (HALO AI) algorithm fine-tuned to our specimens. Subsequently, we developed the DenseNet v2 (HALO AI) classifier to identify fibrin-rich and erythrocyte-rich clot areas (Figure 3). Additionally, we trained the Nuclei Seg (HALO AI) classifier based on cell degradation to ensure correct cell identification.

### 2.6. Statistical Analysis

Continuous variables were expressed as a mean ± standard deviation (SD) or a median and interquartile range (IQR), whereas categorical variables were presented as the number of observations (n) and percentages. Student’s *t*-test and analysis of variance (ANOVA) test (with Tukey’s test for post hoc analysis) were applied for continuous normally distributed data and Mann–Whitney U test was used for continuous data with non-normal distribution. The Shapiro–Wilk test was utilized to evaluate the normality of the data. For categorical data, the chi-square test was used (in cases where at least one expected cell count was <5, Fisher’s exact test was used). Univariate linear regression models were used to analyze the association between clot fibrin proportion and both clinical and radiologic variables (variables with a significance level of *p* < 0.1 were planned to be included in the multivariate model). Statistical significance was set at *p* < 0.05. All statistical analyses were performed using SPSS software (version 28.0; IBM, Armonk, NY, USA).

## 3. Results

The median patient age was 76 years (IQR, 69–82 years), and 43 (54.4%) patients were female. The median admission National Institutes of Health Stroke Scale score was 12 (IQR, 8–16). There were 53 patients (67.1%) with known or newly diagnosed atrial fibrillation. The occlusion site was the distal internal carotid artery in 9 (11.4%) patients, the M1 segment in 37 (46.8%), the M2 in 23 (29.1%), the distal VA (V4 segment) in 4 (5.1%), and the basilar artery in 6 (7.6%). Fifteen patients (19%) received an intravenous bridging recombinant tissue plasminogen activator. The distribution of LAA morphology types was as follows: 24 (30.4%) chicken-wing, 30 (38.0%) windsock, 18 (22.8%) cactus and 7 (8.9%) cauliflower. Table 1 compares the demographic and baseline characteristics of patients with chicken-wing and non-chicken-wing LAA morphologies.

**Figure 4:**
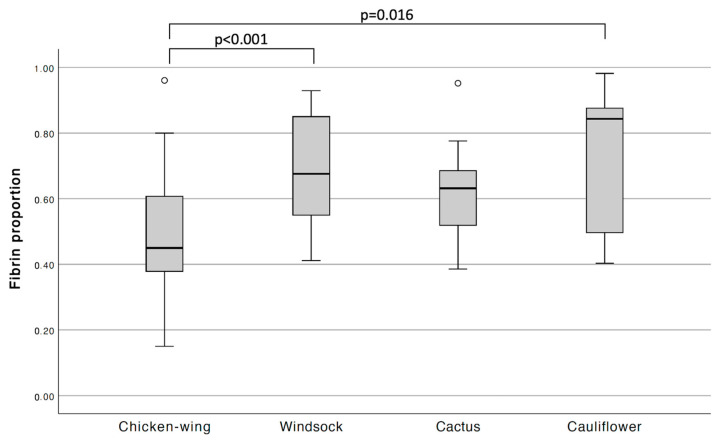
Box plot showing clot fibrin proportion in different left atrial appendage morphologic types. *p* values are provided for post hoc Tukey’s test.

Comparing the LAA types using ANOVA, the clot fibrin proportion was significantly different between LAA types (*p* < 0.001). Post hoc analysis showed lower fibrin content in the chicken-wing-type LAA group than in the windsock-type and cauliflower-type LAA groups (Figure 4). Due to the small sample size for each morphology type, we combined all non-chicken-wing types into one group for further analysis. The chicken-wing-type LAA group had a lower mean clot fibrin proportion than the non-chicken-wing morphology group did (0.49 ± 0.18 vs. 0.67 ± 0.16, *p* < 0.001). There was no significant difference in cell density between the chicken-wing and non-chicken-wing LAA groups (1093 [770–1500] vs. 1236 [665–1700] cells/mm^2^, *p* = 0.639). Also, no significant differences existed between the two groups when comparing the successful recanalization rate (18 [75%] vs. 35 [63.6%], *p* = 0.402) or first-pass effect (12 [50.0%] vs. 25 [45.5%], *p* = 0.710) (Table 2).

In univariate linear regression analysis, only the chicken-wing-type LAA was significantly associated with the clot fibrin proportion (estimate, −0.177; 95% CI [−0.259, −0.096]; *p* < 0.001), while no significant associations were found between the clot fibrin proportion and patient age, gender, presence of atrial fibrillation, arterial hypertension, diabetes mellitus, administration of intravenous thrombolysis and LA and LAA volumes (Table 3).

## 4. Discussion

We explored the association between clot composition and the anatomical LA features in a cohort of patients with embolic stroke. Our findings revealed an association between the chicken-wing LAA morphological type and a lower clot fibrin content. To our knowledge, this is the first study to investigate the relationship between intracranial clot histology and LAA morphology in patients with embolic stroke.

Earlier studies have demonstrated that chicken-wing LAA morphology is associated with a lower stroke and thrombus formation risk, compared with other LAA types [6,14,15,16]. A plausible explanation for our findings could be the larger proportion of other embolic sources in the chicken-wing morphology group. Clot formation sites in these patients could comprise those that are more frequently encountered in cases of ESUS [17], including patent foramen ovale, carotid and vertebral arteries with hemodynamically insignificant (<50%) plaques, cardiac valvular disease, atrial cardiopathy and left ventricular dysfunction.

Another explanation could be the different thrombus formation conditions due to the distinct flow dynamics mediated by the LAA configuration. Studies have described that, in patients with AF, LAA flow velocity is significantly higher in chicken-wing-type LAAs than in other types, and this morphology has a lower spontaneous echo contrast prevalence [18,19,20,21]. In a cohort of patients with sinus rhythm (SR), Shimada et al. demonstrated that chicken-wing morphology was associated with higher volume variability during the cardiac cycle than non-chicken-wing morphology was [22]. Qureshi et al. constructed a flow dynamics model for different LAA morphologies to simulate clot formation during SR and AF [7]. The authors found that during SR, the fibrin gel formed was effectively washed out of LAAs with chicken-wing morphology. However, the fibrin gel was retained for longer in LAAs with windsock and cauliflower morphologies. Models like this could partially explain the over-representation of fresher (and, therefore, RBC-rich) clots in patients with chicken-wing-type LAA, particularly in paroxysmal AF cases. During SR, fresh thrombus material can easily be washed out of the chicken-wing LAA, whereas it may be retained for longer in other LAA types, facilitating organization.

When assessing LAA morphology, there is a substantial variation in the classification methodology used across studies, causing significant differences in the prevalence of each type (e.g., chicken-wing, ranging from 17.5% to 50.9%) and affecting the stroke risk assessment [11,19]. To address this issue, Yaghi et al. suggested a simple classification based on a 90° bend to differentiate between high- (>90°) and low-risk (<90°) LAA types [6]. Our study used a modified classification by Kimura et al. [11], requiring a 100° bend for classification as a chicken-wing. Therefore, the classification was essentially based on the LAA bend angle when comparing the chicken-wing and non-chicken-wing groups in our study, making the morphology discrimination for these two types less susceptible to subjectivity.

The results of this study may have potential implications for clinical practice. The extent of the etiologic workup could be tailored based on the LAA type. Patients with paroxysmal AF and chicken-wing morphology might still undergo additional investigations to identify other potential sources of embolism, adding emphasis on more thorough inspection of the ascending aorta and aortic arch, as well as magnetic resonance imaging (MRI) of the affected artery wall to identify non-stenosing plaques with high-risk features. Secondary stroke prevention could also be modified by adding antiplatelet therapy in patients with chicken-wing morphology and AF, especially in the case of recurrent stroke in patients already receiving anticoagulation.

Our study has some limitations. First, we used non-electrocardiogram-gated chest CTA (as previously described [9,23]), possibly distorting the LA and LAA anatomy due to motion artifacts. Second, the cardiac scan was exclusively performed in the arterial phase, making it difficult to adequately assess LAA lumen for thrombi and trabeculations in patients with circulatory stasis. Third, our histologic analysis measured only the content of RBCs and fibrin, whereas more comprehensive investigations, such as DNA, Von Willebrand factor analysis, neutrophil extracellular trap measurement and immunohistochemical analysis, could provide additional significant data. Fourth, a single-center design and small sample limits the generalizability of our findings. Future larger studies with multicenter designs, incorporating ECG-gated multiphase cardiac CTA, MRI of the affected artery wall, extended cardiac rhythm monitoring, and expanded histological analysis, could provide more robust data on the association between LAA morphology and embolic clot structure.

## 5. Conclusions

In conclusion, this exploratory study provided novel data regarding clot fibrin content variation in distinct LAA types. These findings could potentially have an impact on etiology determination, secondary stroke prevention strategies and the extent of diagnostic investigations. However, further research with multicenter designs, larger patient cohorts and more detailed clot and LAA assessments are needed to confirm the observed association.

## Figures and Tables

**Table 1:** Patient demographic and baseline characteristics.

	Chicken-Wing-Type LAA (*n* = 24)	Non-Chicken-Wing-Type LAA (*n* = 55)	*p* Value
Age, years (median [IQR])	75 (66–82)	76 (69–84)	0.543
Female, *n* (%)	14 (58.3)	29 (52.7)	0.645
NIHSS score on admission, points (median [IQR])	14 (9–18)	12 (8–16)	0.269
ASPECTS on admission, points (median [IQR])	8 (6–9)	8 (7–10)	0.205
Atrial fibrillation, *n* (%)	14 (58.3)	39 (70.9)	0.274
Hypertension, *n* (%)	17 (70.8)	48 (87.3)	0.109
Diabetes mellitus, *n* (%)	4 (16.7)	16 (29.1)	0.243
CTA features			
LA volume, mL (median [IQR])	98.5 (87.3–112.5)	129.5 (95.3–161)	0.067
LAA volume, mL (median [IQR])	11.1 (8.6–15.9)	12 (7.3–17.0)	0.850
LAA thrombus, *n* (%)	0 (0)	6 (10.9)	0.170
LAA stasis, *n* (%)	6 (25.0)	20 (36.4)	0.323
Occlusion site, *n* (%)			
Distal ICA	2 (8.3)	7 (12.7)	–
M1	11 (45.8)	26 (47.3)	–
M2	6 (25.0)	17 (30.9)	–
Basilar artery	3 (12.5)	3 (5.5)	–
Distal VA (V4 segment)	2 (8.3)	2 (3.6)	–
Intracranial clot length, mm (median [IQR])	12 (10–15)	10 (6–15)	0.205
IVT, *n* (%)	5 (20.8)	10 (18.2)	0.764

IQR, interquartile range; NIHSS, National Institutes of Health Stroke Scale; ASPECTS, Alberta Stroke Program Early CT Score; LAA, left atrial appendage; LA, left atrial; CTA, computed tomography angiogram; ICA, internal carotid artery; VA, vertebral artery; IVT, intravenous thrombolysis.

**Table 2:** Histological features and recanalization parameters in the chicken-wing and non-chicken-wing LAA patient groups.

	Chicken-Wing-Type LAA (*n* = 24)	Non-Chicken-Wing-Type LAA (*n* = 55)	*p* Value *
Histological features			
Proportion of fibrin in clot, mean ± SD	0.49 ± 0.18	0.67 ± 0.16	<0.001
Nucleated cells in clot, cells/mm^2^ (median [IQR])	1093 (770–1500)	1236 (665–1700)	0.639
Recanalization			
mTICI score, *n* (%)			
0–2A	0 (0)	5 (9.1)	–
2B	6 (25.0)	15 (27.3)	–
2C–3	18 (75.0)	35 (63.6)	–
No. of passes, median (IQR)	1 (1–2)	1 (1–2)	0.527
FPE, *n* (%)	12 (50.0)	25 (45.5)	0.710

SD, standard deviation; IQR, interquartile range; LAA, left atrial appendage; mTICI, Modified Thrombolysis in Cerebral Infarction; FPE, first-pass effect. * Statistical significance was set to *p* = 0.0125 after Bonferroni correction for multiple comparisons.

**Table 3:** Univariate linear regression analysis for the association between different clinical and radiologic variables and clot fibrin proportion.

Variable	Estimate (95% CI)	*p* Value
Age	0.001 (−0.003, 0.004)	0.726
Female sex	0.016 (−0.068, 0.100)	0.704
Atrial fibrillation	−0.033 (−0.121, 0.056)	0.467
Hypertension	0.080 (−0.028, 0.188)	0.142
Diabetes mellitus	−0.001 (−0.097, 0.096)	0.989
IVT	−0.060 (−0.165, 0.046)	0.265
Chicken-wing LAA	−0.177 (−0.259, −0.096)	<0.001 *
LA volume	<0.001 (−0.001, 0.001)	0.392
LAA volume	−0.001 (−0.009, 0.007)	0.826

CI, confidence interval; IVT, intravenous thrombolysis; LA, left atrium; LAA, left atrial appendage; *, *p* < 0.05.

## Data Availability

Data available on request due to legal restrictions. Please direct inquiries to givi.lengvenis@santa.lt.
